# Direct Observation of Hydroxyls Formed from Water and Oxygen on Ag(100)

**DOI:** 10.1021/acs.jpclett.5c03296

**Published:** 2026-01-11

**Authors:** Cole A. Easton, Sarah M. Stratton, Nima Rajabi, Nishadi Amarathunga, Elizabeth E. Happel, Avery S. Daniels, Adrian Hunt, Hojoon Lim, Vinita Lal, Nipun T.S.K. Dewage, Dennis Meier, Iradwikanari Waluyo, Matthew M. Montemore, E. Charles H. Sykes

**Affiliations:** † Department of Chemistry, 1810 Tufts University, Medford, Massachusetts 02155, United States; ‡ Department of Chemical and Biomolecular Engineering, 5783 Tulane University, New Orleans, Louisiana 70115, United States; § National Synchrotron Light Source II, 8099 Brookhaven National Laboratory, Upton, New York 11973, United States

## Abstract

The interaction of oxygen with silver is a key descriptor of the catalytic reactivity of silver nanoparticles which are ubiquitous in large-scale partial oxidation reactions like ethylene epoxidation. Despite Ag(100) being proposed as the most selective facet, it is less studied than (111) and (110) surfaces. Using scanning tunneling microscopy and synchrotron X-ray photoelectron spectroscopy, we report that, in addition to the well-known O adatoms formed from O_2_ dissociation on Ag(100), hydroxyl groups (OH), at a binding energy of ∼ 531 eV, are also present. The O/OH ratio depends on exposure to water and surface temperature. These assignments are consistent with our density functional theory calculations, which indicate that the formation of two OH groups from an O atom and H_2_O molecule is exothermic. These results indicate that, in addition to O, OH is present even under ultrahigh vacuum conditions and therefore should be considered in proposed catalytic pathways.

## Graphical abstract

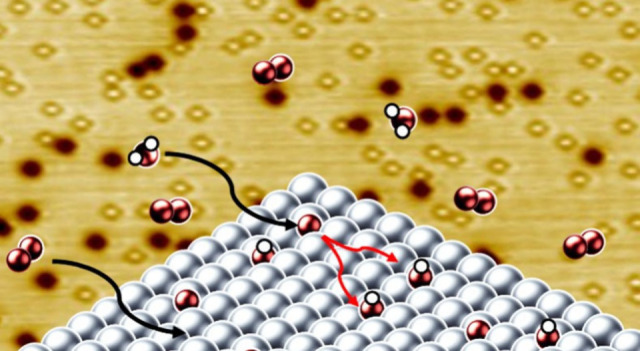

Oxygen on silver is an important catalytic system due to its use in large-scale industrial processes, particularly the partial oxidation of methanol to formaldehyde^1,2^ and the epoxidation of ethylene to ethylene oxide.^3,4^ As a result, extensive research on this system has been performed in order to understand and improve these reactions, especially ethylene epoxidation which accounts for 3% of chemical industry CO_2_ emissions.^5,6^ Despite decades of epoxidation research, key questions remain, namely the state of the surface under reaction conditions, the nature of the selective active oxygen species, and the exact mechanism of selective epoxidation.^6–9^ Furthermore, the interaction of water with oxygen on silver surfaces is relevant in this area due to both the promotional and deleterious effects water can have on heterogeneously catalyzed reactions such as oxidations.^10^ Formed by the reaction of water with surface oxygen, OH species may be present on Ag as well.^11–15^ The majority of experimental studies of OH have focused on Ag(110)^13,16–22^ on which the presence of water and O adatoms leads to OH formation.^13,16,19^ On Ag(100), theoretical studies found that the most stable adsorption site for OH is the 4-fold hollow site, and that OH formation from the interaction of water and preadsorbed oxygen is exothermic.^23,24^ However, the challenge of clearly distinguishing and identifying surface OH in the presence of other adsorbates on Ag surfaces has potentially limited the quantitative experimental work in this area.^10,18,23^

Recently, more attention has been called to the Ag(100) facet^25–34^ due to its inherent selectivity toward ethylene oxide potentially exceeding that of other facets.^35–38^ Additionally, the higher sticking probability of oxygen on Ag(100) enables more opportunities to study this system cleanly in ultrahigh vacuum (UHV), without the need for high-pressure cells.^39,40^ Using X-ray photoelectron spectroscopy (XPS), previous work demonstrated that the introduction of oxygen at room temperature forms two distinct species on Ag(100) at 530 and 531 eV.^25^ In that study, the 530 eV feature was assigned to O in a missing-row surface reconstruction and the 531 eV feature to subsurface O.^25^ In studies on other facets, the 530 eV peak in O 1s spectra on O–Ag surfaces at low coverages is generally assigned as atomic oxygen,^41–43^ while there is considerable debate over the nature of the 531 eV species. Various O 1s peaks at ∼ 531 eV have been attributed to several forms of oxygen such as carbonate,^44^ molecular oxygen,^45^ hydroxyls/hydroxides,^46,47^ and subsurface/bulk oxygen.^25,48–50^ In order to evaluate and determine the identity of this higher binding energy peak, we correlated XPS and scanning tunneling microscopy (STM) experiments with density functional theory (DFT) calculations. Together our work provides strong evidence that the 531 eV peak observed in XPS arises from OH species that form spontaneously when water interacts with the O–Ag(100) system. Even the typical UHV conditions contain enough background water for OH to form; therefore, one must consider this species as a likely surface species under reaction conditions.

In order to study the surface species present after Ag(100) is exposed to molecular oxygen, we first performed STM experiments at 78 K. Specifically, we exposed a clean Ag(100) surface to 1,000 Langmuir (L) (1 L = 1 × 10^–6^ Torr·s) O_2_ at 300 K, which resulted in the formation of two species observed with STM (Figure 1A). These species appear as isolated depressions (18–20 pm depth, red arrow) and isolated protrusions (3–5 pm height, blue arrow). The depressions are consistent with previous reports of O adatoms formed by the dissociative adsorption of O_2_ on Ag(100),^27,28,51^ but the identity of the other species appearing as a protrusion was initially unclear. Subsequent XPS measurements (Figure 1B,C) also demonstrated the presence of two species, one at a binding energy of 530 eV, which has previously been assigned to O adatoms (red XPS peak),^41–43^ and a second peak at 531 eV (blue peak), which has been assigned in the literature to a number of species that include subsurface oxygen, hydroxyl species, sulfate, molecular oxygen and carbonate.^7,25,44–50^ As seen in the XPS spectra in Figure 1B, annealing the surface to 400 K reduced the size of the 530 eV peak, but almost completely removed the signal at 531 eV: as shown quantitatively in Figure 1C. The appearance of the oxygen feature as a depression in STM, as well as the behavior of the lower binding energy O 1s peak at 530 eV, are consistent with this species being O adatoms.

**Figure 1:**
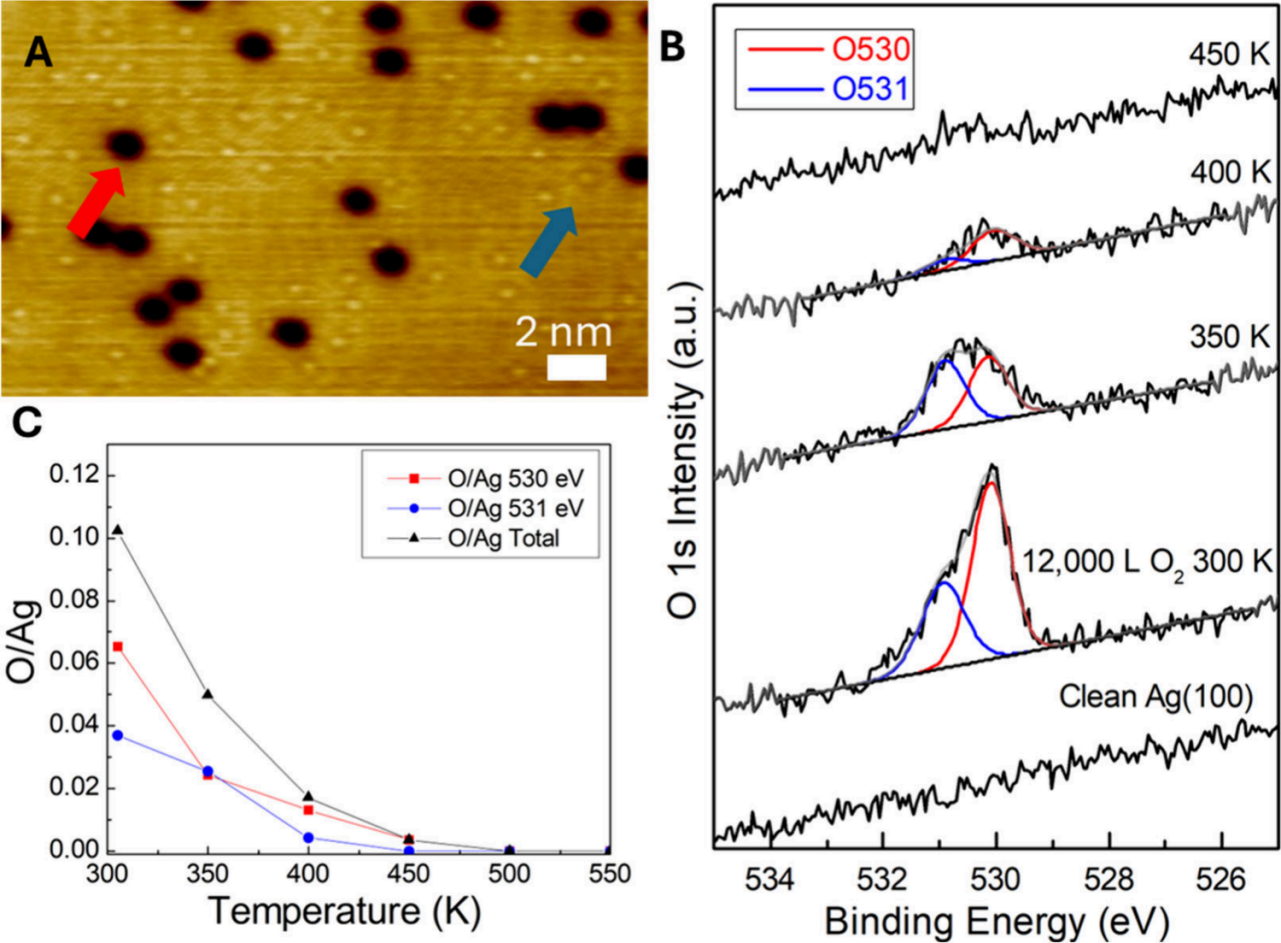
Products resulting from O_2_ dissociation on Ag(100). (A) 78 K STM image of ∼ 1% ML depressions (red arrow) and ∼ 1.5% ML protrusions (blue arrow) resulting from a 1000 L dose of O_2_ on Ag(100) at room temperature. Imaging conditions: 10 mV, 1 nA. (B) O 1s XPS spectra of clean Ag(100), 12,000 L O_2_ 300 K (7% O530 and 4% O531) on Ag(100), and subsequent anneal. (C) Plot of the coverage of each O 1s component from the fits in A.

To determine the identity of the 531 eV species, STM, XPS and DFT were used to narrow down the number of potential candidates. DFT calculations in which O atoms were placed in octahedral and tetrahedral subsurface sites under one Ag layer revealed that any form of subsurface oxygen would be ∼ 2 eV less stable than on the surface (Figure S1), which agrees with other DFT studies on Ag(100), thus ruling out subsurface oxygen.^52–54^ Additionally, several attempts were made to stabilize O in the subsurface below the 4-fold hollow site, which all resulted in the O spontaneously relaxing to the surface.

Molecular O_2_ was ruled out using control experiments in which O_2_ was deposited on the sample at 78 K in the STM stage. These experiments indicated that molecular oxygen is not visible in STM images because of its high mobility and weak binding,^55^ which leads to fast diffusion and the inability to be imaged.^29^ Importantly, during these adsorbed O_2_ control experiments, the two species shown in Figure 1A were not observed. Sulfur, a common impurity in silver samples, was also ruled out by using XPS to check the sample for sulfur, and by using STM to demonstrate that the observed protrusions do not have the same known bias dependence as sulfur atoms (Figure S2).^56^ Similarly, carbonate was ruled out using XPS, which shows the absence of any carbon signal in the C 1s region (Figure S2).

This left OH as the most likely candidate for the observed species. The XPS binding energy of OH on Ag has been reported in the range of 530 to 533 eV, consistent with our 531 eV assignment.^17,18,46,47^ ^,^ ^57–59^ Furthermore, our DFT calculations predict a low barrier (0.17 eV) for an oxygen adatom reacting with water to form adsorbed OH groups (O + H_2_O → 2OH), as well as a favorable reaction energy (−0.56 eV) (Figure 2A). Combined, these data support OH being the species observed as isolated protrusions in the STM images and the 531 eV peak in XPS. After O_2_ is dosed at 300 K, background water reacts with surface oxygen, leading to the formation of OH groups and a reduction of the number of O adatoms as seen in Figure 2. Further evidence for this role of background water comes from observations of fewer OH species directly after a full UHV chamber bakeout at 140 °C.

**Figure 2:**
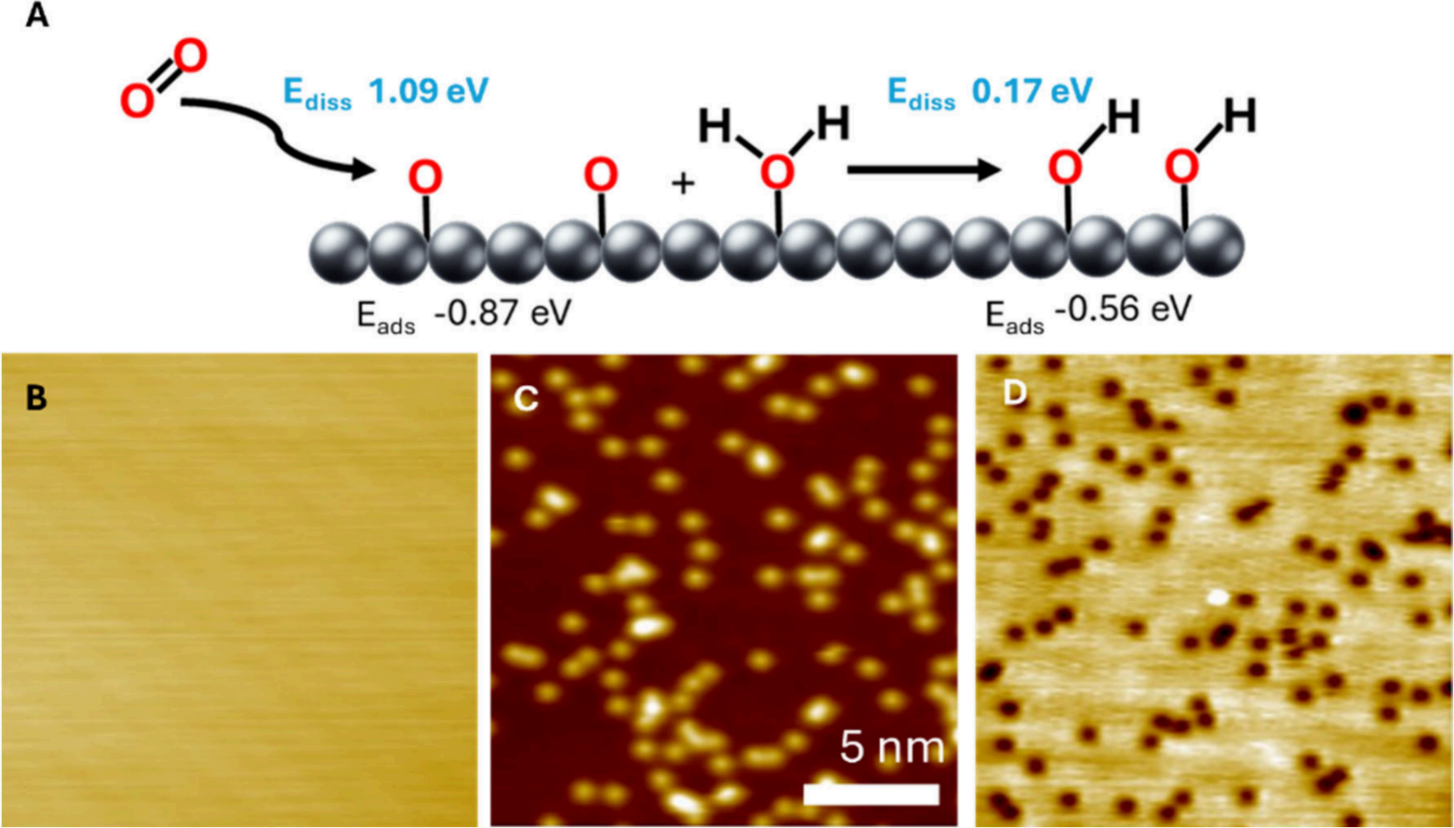
78 K STM images of the Ag(100) surface before and after exposure to O_2_ under different imaging conditions. (A) Depiction of oxygen dissociation and reaction with water on the Ag(100) surface and associated reaction energies and activation barriers. Dissociation barriers are shown in blue (E_diss_), and adsorption energies are shown beneath in black (*E*_ads_). All energy values are relative to gas phase O_2_. (B) 78 K STM image of the bare Ag(100) surface before exposure to O_2_. Imaging conditions: 300 mV, 300 pA. (C)-(D) Images of O adatoms (depressions) and OH groups (protrusions) on Ag(100) imaged at (C) 3.00 V, 300 pA, (D) 300 mV, 300 pA. (C) and (D) are of the same area. At 3.00 V, only protrusions are visible, and at 300 mV only depressions are visible. Surface coverages were 1.0% O and 1.5% OH in (C) and (D). All images are 50 × 50 nm^2^, scale bar shown in C.

STM experiments also indicated that the appearance of the two species was highly dependent on the imaging conditions, and under some conditions only one of the species is visible. Specifically, scanning at a bias of 3.00 V leads to only protrusions being visible (Figure 2C), while scanning between 100 mV and 1.00 V leads to only depressions being visible (Figure 2D). The depressions appear in different locations than the protrusions, which rules out an inversion effect. The tunneling current did not significantly affect the appearance of either species. However, imaging of both species concurrently and discretely is only possible at conditions of ∼ 10 mV and ≥ 1 nA (Figure 1A). As a result, most imaging was performed at 10 mV, 1 V, or 3 V bias to selectively image either or both species. Selective imaging at the specified conditions allows for easier visualization and surface coverage quantification of the two species. Negative sample biases did not exhibit the same effects (Figure S3). The bias-dependent signatures of the individual species, together with DFT-based STM image simulations, further confirm their identities. Specifically, both the experimental STM images (Figure S3) and the DFT simulated STM images (Figure S4) show that OH consistently appears as a protrusion at all STM imaging conditions. In contrast, O adatoms appear as depressions at lower voltage biases but as protrusions at higher biases (>4 V). These bias-dependent imaging behaviors therefore support assigning the protruding 531 eV feature to OH.

In order to examine the adsorption site of the O and proposed OH species, we performed high-resolution STM imaging (Figure 3). Images of the O adatom show it binding in the 4-fold hollow site of Ag(100) (Figure 3A, B). This adsorption site has been widely shown to be the preferred site for O adatoms on Ag(100),^5,52,53^ but has not been imaged with atomic resolution before.^27,28,51^ This assignment is also consistent with our DFT calculations, which indicate that oxygen atoms bind in 4-fold hollow sites on Ag(100) (Figure 3E). Figure 3C shows the protrusion and depression features imaged in the same frame, and the insets show simulated STM for the appearance of OH (top right) and O (lower), which agrees with our assignments.

**Figure 3:**
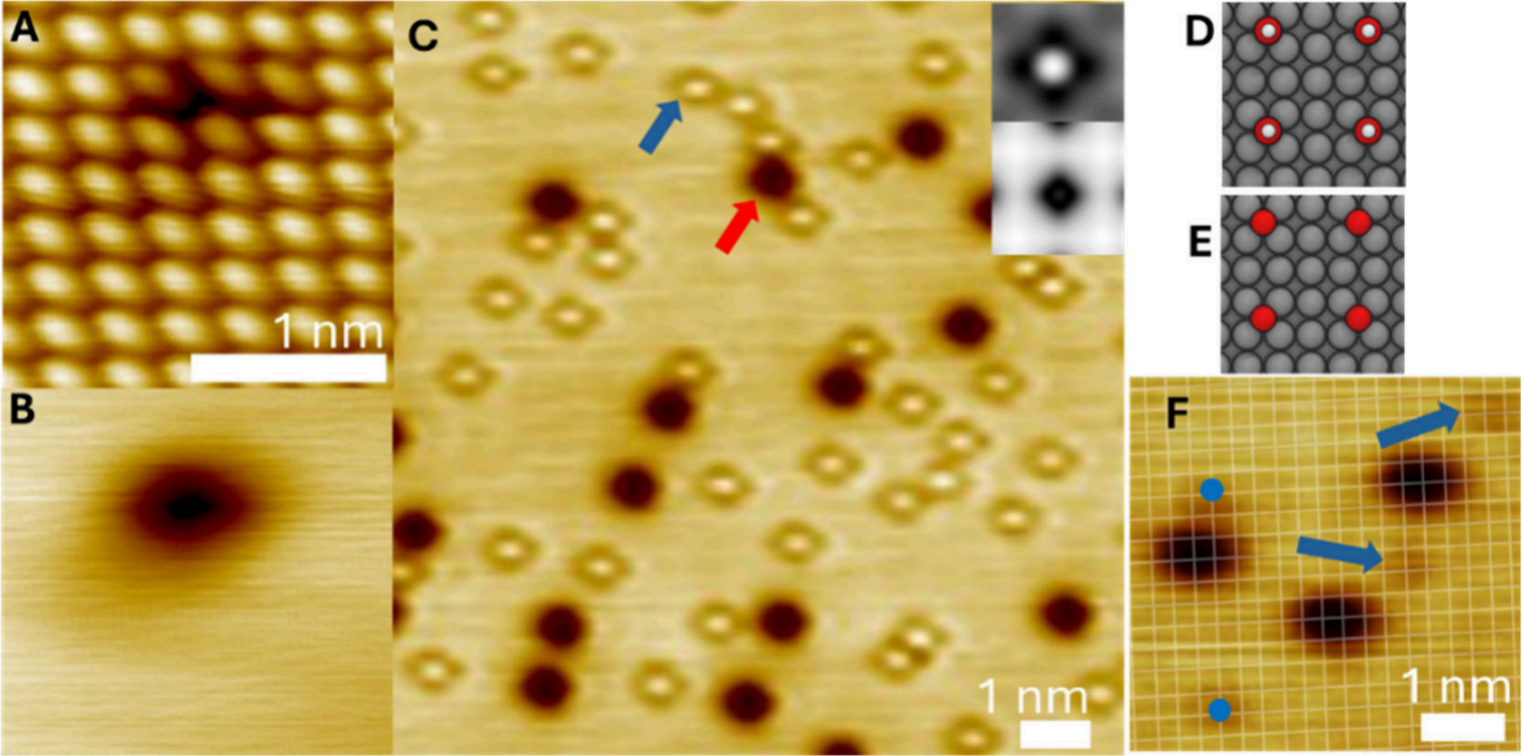
Binding site characterization of O adatoms and OH groups on O–Ag(100). (A) Atomic resolution 78 K STM image of an oxygen adatom on Ag(100) imaged at 10 mV, 1 nA. (B) The same area of the surface under typical resolution imaged at 100 mV, 1 nA. Both STM images are the same size, and a scale bar is shown in (A). (C) STM image of O and OH on Ag(100) imaged at 25 mV, 1 nA, and a scale bar is shown. Insets in panel C are (top) Simulated STM image of an OH group on Ag(100), (bottom) simulated STM of an O adatom on Ag(100). (D) and (E) are the calculated preferred binding sites for (D) OH and (E) O atoms. (F) STM image of O and OH with the Ag(100) 4-fold hollow lattice corrected for drift overlaid. The centers of several OH groups are marked with blue circles or arrows. The locations where the grid lines cross are the 4-fold hollow sites. Imaging conditions: 25 mV, 1 nA, and a scale bar is shown. Images were acquired at 78 K.

With the knowledge that the O adatom depressions are all in 4-fold hollow sites, we can overlay a grid of the underlying (100) lattice on a representative STM image and subsequently determine the binding site of the species imaged as protrusions (Figure 3F). As shown in Figure 3F, the protrusions also occupy 4-fold hollow sites, which agrees with the DFT-predicted binding site for OH (Figure 3D).

Localized STM voltage pulsing further supported the identification of the protrusions as surface hydroxyls. This experiment was performed by moving the tip over the target species and then applying a higher voltage bias.^60^ Figure 4 shows representative results of the voltage pulse experiments for protrusions (OH) as well as molecularly adsorbed O_2_ for comparison. Figure 4B and C show STM images before and after pulses were performed over the OH species. All protrusions (OH) from 4B were converted to depressions (O). Figure 4A depicts this process in which a 4 V pulse acts to abstract the H atom from the OH group, forming an O adatom. This experiment was attempted using negative sample biases as well, but no dissociation was observed. Furthermore, performing the same pulses on the O adatoms led to no change.

**Figure 4:**
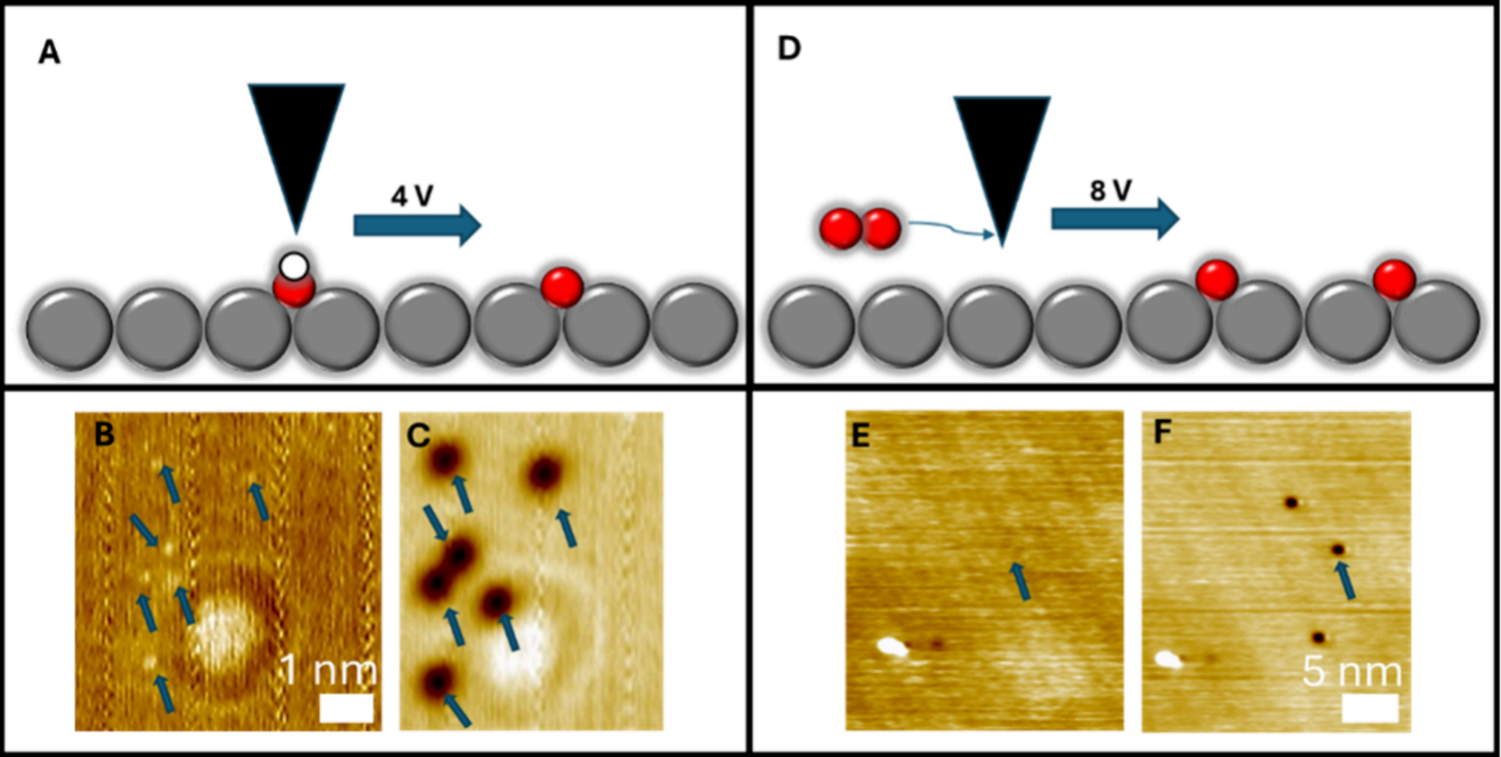
STM Voltage Pulse Experiments. (A) Depiction of STM tip dissociating OH into O and H using a localized voltage pulse. (B) 78 K STM image of the Ag(100) surface before voltage pulses. OH groups appear as faint protrusions and are marked by blue arrows. (C) 78 K STM image of the same area after 4 V pulses. Each OH is now a depression in the same surface location. Oxygen was dosed at 300 K, and the pulse locations are indicated by the blue arrows. (D) Depiction of the STM tip dissociating O_2_ into two O adatoms via a voltage pulse. (E) LT-STM image of the Ag(100) surface with oxygen dosed at 78 K. Oxygen is present as molecular oxygen and diffuses too fast at 78 K to be imaged. The pulse location is indicated by the blue arrow. (F) LT-STM image of the same area following one 8 V pulse. Oxygen adatoms have formed as a result of the pulse. No further changes occurred after pulsing on the O adatoms. Imaging conditions: 1.0 nA and 50 mV.

Conversely, Figures 4E and F show the effects of STM tip pulsing on an O_2_ covered surface and formation of O adatoms. When dosed at 78 K, molecular oxygen adsorbs intact on Ag(100).^28,61^ As noted earlier, when scanning at 78 K, oxygen molecules diffuse too fast to be imaged.^29^ However, the oxygen adatoms formed from positive sample voltage pulses have a higher diffusion barrier and can be imaged at this temperature (Figure 4F).^62^ This comparison further supports our assignment of the OH species and rules out molecular oxygen as the identity of the protrusions.

In order to understand the conditions necessary for the formation of the OH species, we performed STM experiments on O adatoms on Ag(100) before and after the addition of water (Figure 5). Specifically, 1000 L O_2_ was dosed onto a clean Ag(100) crystal at 300 K, and the sample was cooled to 78 K for imaging. The coverages of both O and OH were determined using our species-specific STM imaging conditions (Figure 5A and B). Using our XPS data for guidance, in which the 531 eV peak assigned to OH disappears almost entirely upon annealing while the 530 eV peak remains, the crystal was annealed to ∼ 400 K (5C and D). After annealing, STM imaging reveals that the surface was occupied solely by O adatoms. This result provides further confirmation of the assignment of O and OH species based on their apparent height in STM as discussed earlier. Then, 3 L H_2_O was dosed at 78 K. Upon the introduction of water, we observed the formation of more protrusions (OH) and a decrease in depressions (O adatoms) (5E and F). The surface coverages for each step of the experiment are summarized in Figure 5G. The formation of protrusions from the introduction of water and subsequent decrease in O adatoms indicates that when water is introduced, O adatoms react with a water molecule to produce two OH groups. As a control, water was dosed onto a bare Ag(100) surface at 78 K, and no features (protrusions nor depressions) were observed (Figure S8). Together, these multiple XPS and STM experiments, coupled with DFT predictions, provide strong evidence that, in addition to the well-known oxygen adatoms on Ag(100) that result from room-temperature dissociative adsorption of O_2_, OH species are present and appear in XPS with a binding energy of 531 eV. These OH groups form from the reaction of water with surface oxygen atoms and can be removed from the surface by annealing to 400 K.

**Figure 5:**
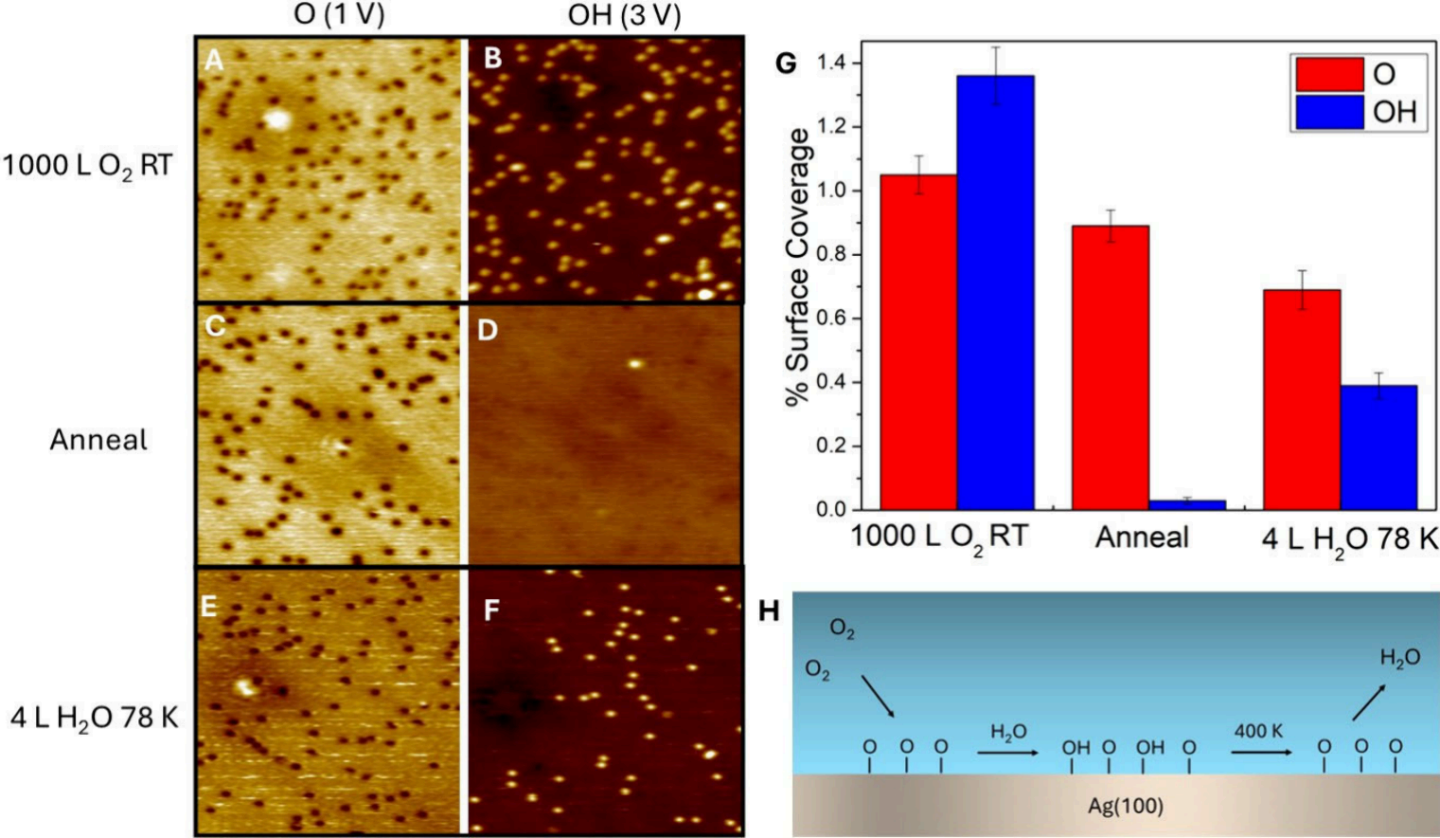
Reaction of O atoms with adsorbed H_2_O. (A) 78 K STM image of 1000 L O_2_ dosed at 300 K on Ag(100) imaged at 1 V. O adatoms are visible as depressions. (B) Same area imaged at 3 V where OH groups are visible as protrusions. (C) STM image of O–Ag(100) surface after a 400 K anneal scanned at 1 V. (D) Same area scanned at 3 V showing a significant reduction in the number of OH species. (E) STM image of the surface after 3 L H_2_O was dosed at 78 K, annealed to 400 K, and then imaged at 1 V at 78 K. (F) Same area imaged at 3.0 V showing an increase in the number of OH species. (G) Quantification of the surface coverages of O and OH for each stage of the experiment. (H) Schematic summarizing O_2_ dissociation, OH formation, and water desorption from Ag(100). Tunneling current was constant for all STM images at 300 pA. All images are 50 × 50 nm^2^.

In summary, this study provides direct atomic-scale evidence that hydroxyl (OH) species form spontaneously on Ag(100) surfaces through the reaction of adsorbed oxygen with trace water, even under ultrahigh vacuum conditions. Under these conditions, two oxygen-related surface species are observed and assigned to atomic O adatoms at a binding energy of 530 eV and OH groups at 531 eV. Consistent with DFT predictions, STM imaging shows that both species occupy 4-fold hollow sites, with OH appearing as protrusions and O adatoms as depressions depending on imaging bias. STM voltage-pulsing experiments reveal that OH can be converted to O via hydrogen abstraction. These findings shed light on the long-standing ambiguity surrounding the 531 eV oxygen peak and demonstrate that surface hydroxyls, previously overlooked, are likely ubiquitous on Ag surfaces and should therefore be considered in proposed reaction pathways and models of partial oxidation reactions.

## Experimental Methods

78 K STM experiments were conducted in an ultrahigh vacuum (UHV) chamber with a base pressure of 1 × 10^–11^ mbar using a low temperature (LT)-STM (Omicron Nanotechnology). Two Ag(100) single crystals (Princeton Scientific) were used for experiments. Sample cleaning was performed in a connected preparation chamber with a base pressure of <5 × 10^–10^ mbar. Repeated cycles of Ar^+^ (Airgas 99.99%) ion sputtering (1 keV 10–20 μA) and annealing to 825 K were used to clean the Ag(100) crystals. STM images were obtained at 78 K after cryogenically cooling the STM stage. Oxygen (99.9% Middlesex gases) and water (Fisher Scientific HPLC grade 99%) were dosed through high-precision leak valves. Coverages were calculated as an average from ∼ 20 images for each coverage through manual counting of features using an STM image processor (SPIP). Coverages are based on the number of specific features per image divided by the total number of Ag surface atoms in each image. Error bars on coverage were one standard deviation.

XPS experiments were performed at beamline 23-ID-2 (IOS) of the National Synchrotron Light Source II (NSLS-II) at Brookhaven National Laboratory.^63^ All experiments were performed on a Ag(100) crystal cleaned with Ar^+^ ion sputtering and annealing to 825 K until XPS spectra showed no impurities. The heating rate for cleaning and annealing was 40 K/min. High purity O_2_ (Matheson, 99.994%) was exposed to the sample via a precision leak valve while the sample was heated with a pyrolytic boron nitride heater. Temperature was measured with a K-type thermocouple between the Ag(100) crystal and the heater/sample mount. Reference Ag 3d spectra were taken after acquiring each oxygen spectrum. Oxygen species coverages were calculated by dividing the corrected O 1s peak areas by the corresponding corrected Ag 3d peak areas.

DFT calculations were performed with the VASP code^64,65^ and the PBE exchange-correlation functional. A 400 eV energy cutoff was used for the plane-wave basis set. The projector-augmented wave method^66^ was used for core electrons, and the Tkatchenko-Scheffler method was used for dispersion corrections.^67^ A 7 × 7 × 1 k-point grid was used for the 3 × 3 × 1 supercell. Four layers of Ag atoms were used, with the bottom two fixed at their bulk positions. A geometric convergence criterion of 0.03 eV/Å was used, along with an electronic convergence criterion of 10^–5^ eV. Transition state calculations were performed using the dimer method,^68,69^ and vibrational calculations confirmed that the final structure contained one significant imaginary frequency corresponding to the reaction coordinate.

## Supplementary Material



## Notes

Details of experimental methods and additional STM, DFT and XPS data (PDF)
