# Supplementary material for: Direct Observation of Hydroxyls Formed from Water and Oxygen on Ag(100)
Source: J Phys Chem Lett. 2026 Jan 11;17(3):833–40. doi: 10.1021/acs.jpclett.5c03296 (PMC12961951; doi:10.1021/acs.jpclett.5c03296)
Supplement: Supplementary file 1 [file jz5c03296_si_001.pdf]

Supporting Information

Direct Observation of Hydroxyls Formed from Water and Oxygen on Ag(100)

Cole A. Easton,[1] Sarah M. Stratton,[2] Nima Rajabi,[1] Nishadi Amarathunga,[1] Elizabeth E. Happel,[1] Avery S. Daniels,[1] Adrian Hunt,[3] Hojoon Lim,[3] Vinita Lal,[1] Nipun T.S.K. Dewage,[1] Dennis Meier,[1] Iradwikanari Waluyo,[3] Matthew M. Montemore,[2*] and E. Charles H. Sykes.[1*]*

[1]Department of Chemistry, Tufts University, Medford, Massachusetts 02155, United States

[2]Department of Chemical and Biomolecular Engineering, Tulane University, New Orleans, Louisiana 70115, United States

[3]National Synchrotron Light Source II, Brookhaven National Laboratory, Upton, NY, 11973, United States

*charles.sykes@tufts.edu, *mmontemore@tulane.edu, *iwaluyo@bnl.gov

Experimental Methods

X-Ray Photoelectron Spectroscopy (XPS) Analysis

Oxygen species coverages were estimated by dividing the corrected O 1s peak areas by the corresponding corrected Ag 3d peak areas. Peak areas were corrected by dividing the raw areas by their Relative Sensitivity Factor (RSF) value[2] multiplied by the inelastic mean free path (IMFP) of the ejected electron found from the universal curve[3] (Equation S1). Ag 3d peak areas were also corrected by a first layer correction based on the IMFP to account for the signal only generated for the top level of atoms (Equation S2).

$$\text{Corrected O 1s Peak Area} = \frac{\text{Raw Peak Area}}{\text{R.S.F.} \times \text{I.M.F.P.}}$$

Equation S1. O 1s XPS Correction.

$$\text{Corrected Ag 3d Peak Area} = \frac{\text{Raw Ag3d}_{5/2}\text{ Peak Area} \times \text{First Layer Correction}}{\text{R.S.F.} \times \text{I.M.F.P.} \times \text{5/2 Correction}}$$

Equation S2. Ag 3d XPS Correction.

To investigate different elements, different photon energies were used. Predominantly, 760 and 500 eV were used to generate photoelectrons with kinetic energies of ~200-300 eV for the O 1s (at 760 eV) and S 2p and C 1s (at 500 eV) core levels, respectively, to maintain similar probing depths. The latter two elements were only monitored for impurities. Binding energy calibration was performed on all spectra with respect to the Fermi edge and metallic Ag $3d_{5/2}$ binding energy measured at each photon energy.[4] XPS data was analyzed with CasaXPS. Each species was assigned and fit according to experimental results and literature values (Table S1). O 1s spectra were fit with a linear background due to their small size, while Ag spectra were fit with a Shirley background.[5]

**Component**	**Binding Energy (eV)**	**Full Width at Half Maximum (FWHM)**	**Line Shape (Gaussian/Lorentzian)**
Atomic O[2,5–7]	530.0 - 530.1	0.8 – 1.0	30:70
OH[8–10]	530.9 - 531	0.8 – 0.9	30:70
Metallic Ag[4,11]	368.2	0.4	99:1

Table S1. Summary of fitting parameters for XPS spectra. Symmetric peak shapes were used for all fit peaks.

Note on differing O:OH Ratios: We found that OH formation was highly dependent on the amount of oxygen dosed, sample temperature and amount of water in the chamber background. As a result, we see variations in the amount of surface OH present in different UHV chambers and even between experiments in the same UHV chamber as the water background changes.

Supplementary Figures

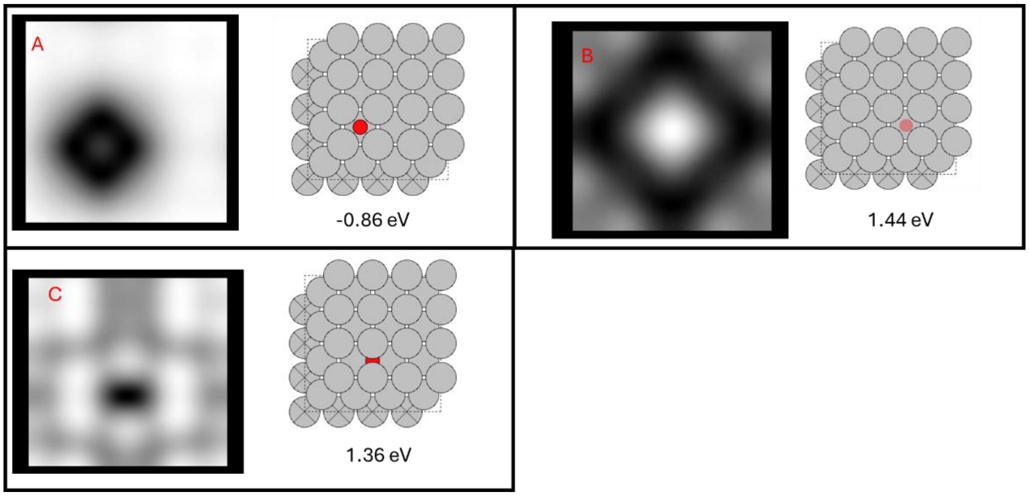

Figure S1. Simulated STM images of surface and subsurface atomic oxygen species on Ag(100), along with DFT-calculated adsorption energies relative to gas-phase O_2. A) Simulated STM image of an oxygen adatom sitting in a four-fold hollow site and associated depiction of said site. B) Simulated STM image of an oxygen atom in a subsurface octahedral site. C) Simulated STM image of an oxygen atom in a subsurface tetrahedral site.

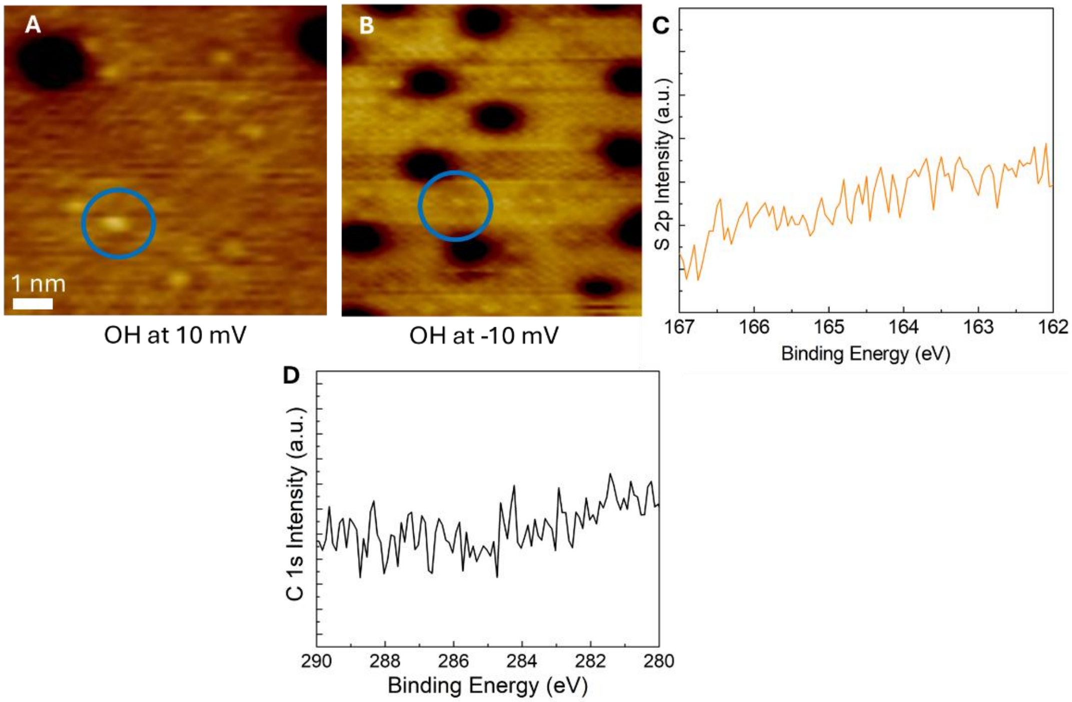

Figure S2. XPS and STM evidence against the presence of sulfur (A-C) and carbonate (D) being associated with the 531 eV feature in the O 1s spectra. A) When OH groups are imaged by 78 K STM with a positive sample bias they appear as donut-shaped protrusions. Imaging conditions are +10 mV and 1 nA. B) STM image of OH groups imaged with a negative sample bias. The OH groups are again present as donut-shaped protrusions. Imaging conditions are -10 mV and 2 nA. The OH groups do not exhibit the bias-dependence typically seen for adsorbed S atoms which appear as protrusions at positive sample voltage and depressions at negative sample voltage.[12] C) XPS scan of the S 2p region taken at 500 eV before experiments on Ag(100) demonstrating there were no S-containing impurities present in the sample. D) XPS scan of the C 1s region taken during the O_2 uptake. The lack of C 1s signal suggests there are no C impurities relevant to these experiments, and thus the presence of carbonate can be ruled out.

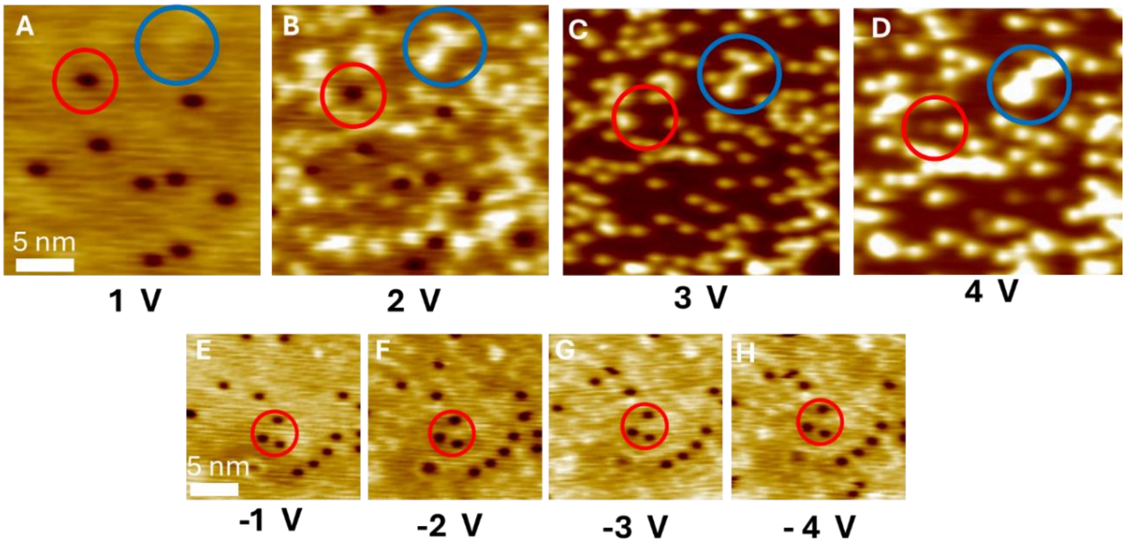

Figure S3. Bias-dependent STM appearance of O-Species. A) 78 K STM image of the O-Ag(100) surface taken at 1.00 V. Only O adatoms are visible as depressions (red circle). B) The same area was imaged at 2.00 V. O adatoms are still visible (red circle) as well as faint OH protrusions (blue circle). C) The same area was imaged at 3.00 V. O adatoms are no longer visible and OH groups are clearer and more discrete. D) The same area was imaged at 4.00 V. O adatoms are visible as dim protrusions and OH groups are visible as bright protrusions (blue circle). All images were taken at 300 pA and a scale bar shown in A. E-H) Equivalent of A-D at negative voltage biases. There is not much voltage dependent change in appearance as with the positive bias regime; OH groups are not visible, and O adatoms retain their appearance as depressions. All images were taken at 300 pA.

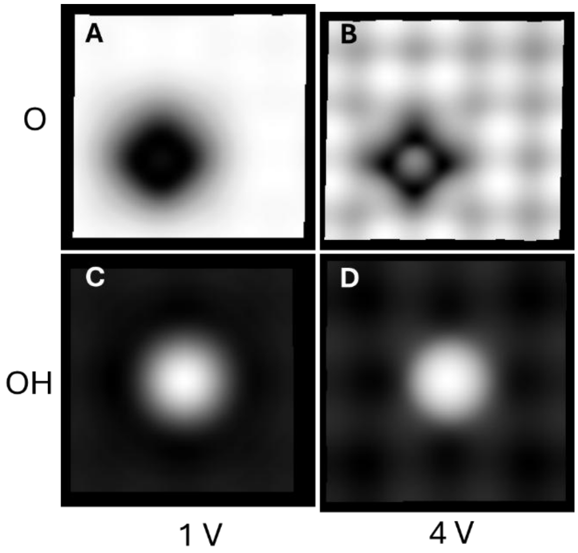

Figure S4. Simulated STM image of an O adatom visible as both a protrusion and a depression at different sample voltages. A) Simulated STM image of an O adatom on Ag(100) at +1 V showing O as a protrusion. B) Simulated STM image of an O adatom on Ag(100) at + 4 V showing O as a depression. C-D) Simulated STM images of OH under the same conditions. OH remains a bright protrusion.

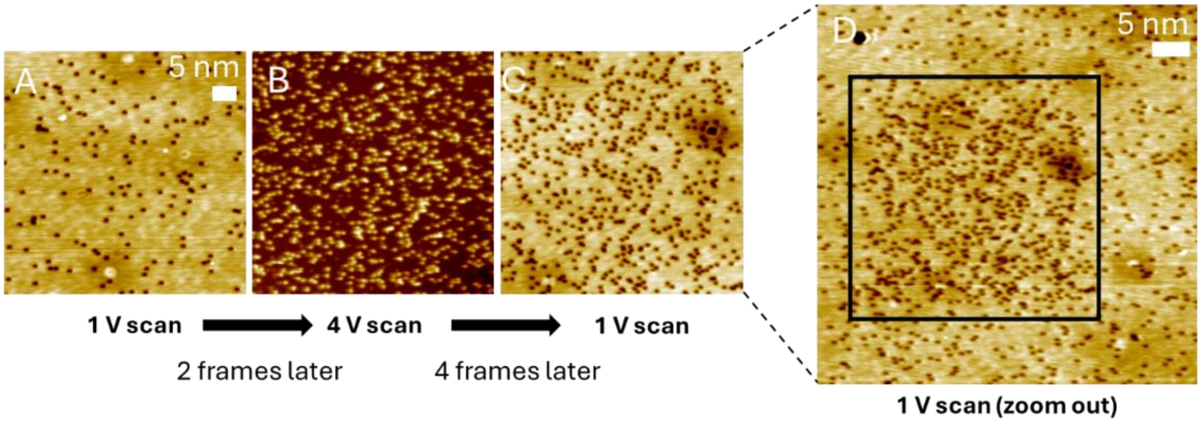

Figure S5. Formation of O adatoms formed by scanning at 4 V. A) STM image of 1000 L O_2 dosed at 300 K on Ag(100). Scan was taken at 1.00 V. O adatoms are visible as depressions. B) The same area was scanned at 4.00 V. OH groups are visible as bright protrusions and oxygen adatoms are visible as dimmer protrusions. C) The same area was scanned at 1.00 V again after 4 frames at 4.00 V. Only oxygen adatom depressions are visible but they have increased in amount/coverage meaning that OH groups were converted to O adatoms by the STM tip at a 4.00 V bias. D) Zoom out of the area shown in A-C. The part of the surface scanned at 4.00 V has a higher oxygen adatom surface coverage (black square) than the surrounding areas due to the voltage-induced conversion of the OH species to O. All images were taken at 300 pA at a temperature of 78 K. Scale bars are shown in A and D.

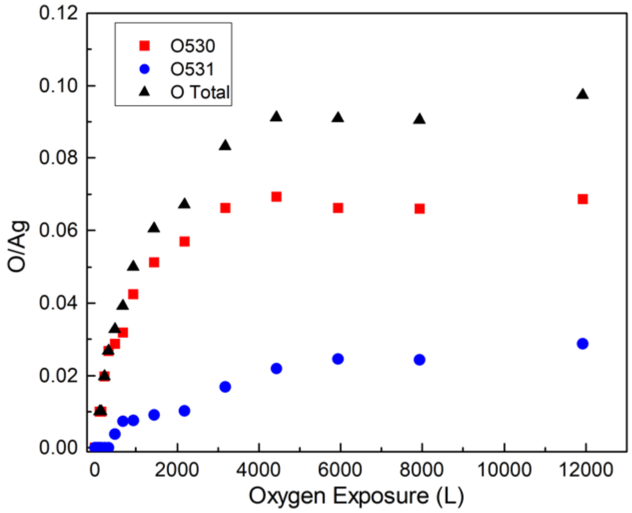

Figure S6. XPS-derived oxygen uptake on Ag(100) at 300 K. Oxygen coverages for the uptake of 12,000 L O_2 dosed at room temperature. Two species are present at 530 eV (red) and 531 eV (blue). The 530 eV peak grew in first, appearing after 70 L O_2 exposure, and the 531 eV peak appears after ~450 L. O 1s spectra were measured at a photon energy of 760 eV.

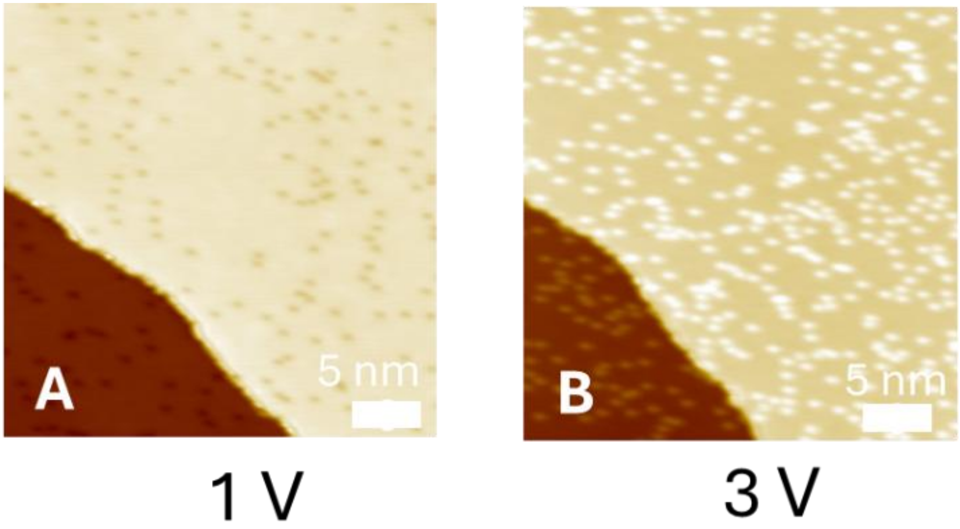

Figure S7. 78 K STM images of O and OH demonstrating the lack of clustering of O adatoms or OH groups at step edges A) 1 V image of an Ag(100) step edge after being exposed to 1000 L O_2 dosed at room temperature. O adatoms are visible as depressions on the upper and lower terraces and, there is no formation of any areas of a higher O or OH density directly above or below the step edge. B) 3 V image of a Ag(100) step edge on the same area as A. Only OH protrusions are visible. There is no evidence of the formation of brims of OH above or below the step edge. Both images are 50 x 50 nm^2 (scale bars are shown in both images) and tunneling current was 300 pA.

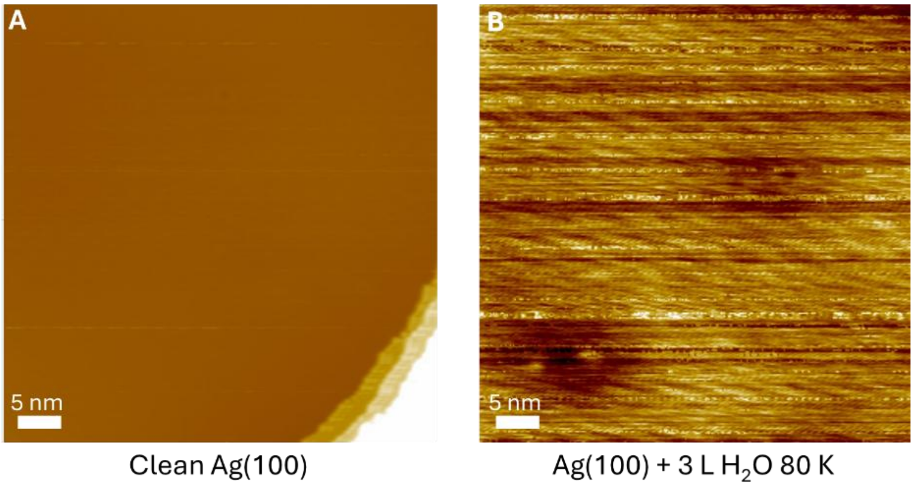

Figure S8. STM before and after H_2O exposure to clean Ag(100) as a control experiment. A) STM image of a clean Ag(100) surface. B) STM image of the same surface after exposure to 3 L H_2O at 78 K. The enhanced level of noise in this STM image is due to physisorbed water molecules interfering with the STM tip while scanning. No new features are present. Images were taken at 78 K. Imaging conditions were 300 mV and 300 pA. A scale bar is shown in both images.

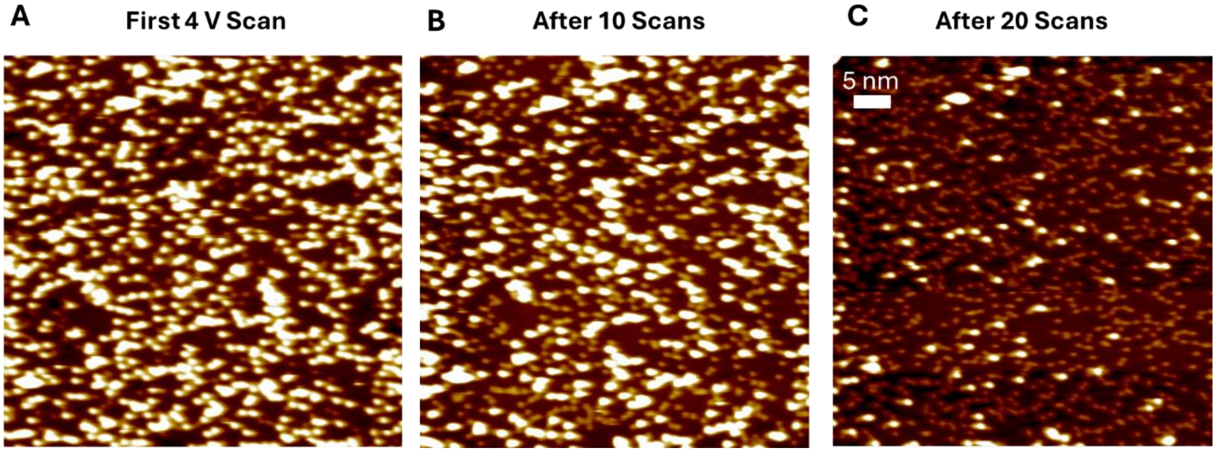

Figure S9. STM images showing the conversion of OH to O over time while scanning at high voltage. A) Initial 4 V scan of Ag(100) after exposure to 1000 L of oxygen at room temperature. OH groups are visible as bright protrusions and oxygen adatoms are visible as dimmer protrusions. B) The same area after 10 scans at 4 V. Many of the OH groups have been converted into O adatoms. C) The same area after 10 additional scans, for a total of 20 scans. The majority of OH groups have now been converted into O adatoms which appear as small protrusions under these imaging conditions. Tunneling current for all images is 300 pA, the sample temperature was kept at 78 K, and all images are 50 x 50 nm^2 (scale bar is shown in C).

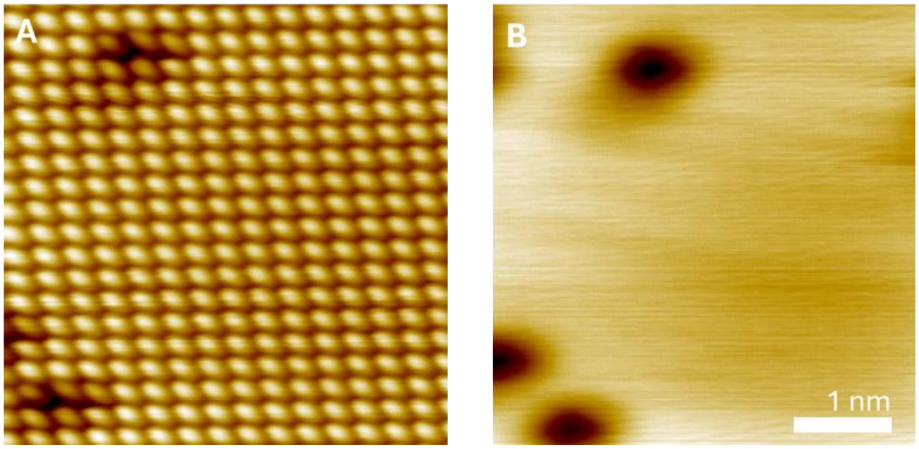

Figure S10. Larger scale atomic resolution STM imaging of O adatoms. A) 78 K STM image of oxygen adatoms on Ag(100) under atomic resolution imaged at 10 mV and 1 nA. B) The same area under typical resolution imaged at 100 mV and 1 nA. A scale bar is shown in B.

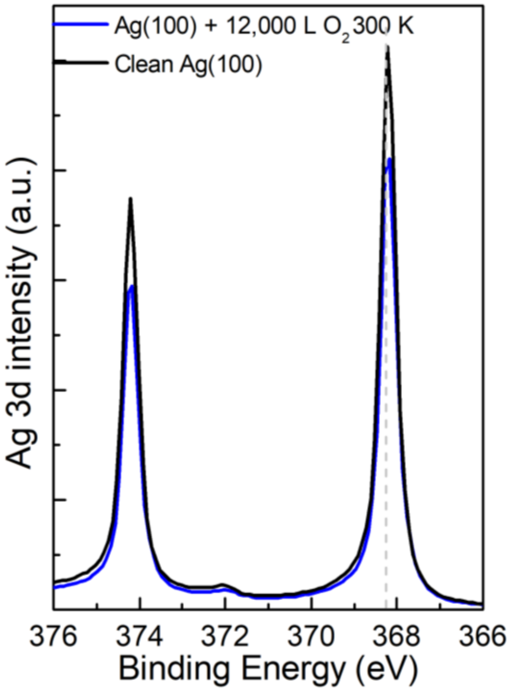

Figure S11. Silver XPS. Ag 3d core level spectrum of clean Ag(100) (black trace) overlaid with the Ag 3d core level spectrum after 12,000 L O_2 exposure on Ag(100) (blue trace). The Ag(100) sample and surface remain metallic (Ag $3d_{5/2}$ peak at 368.2 eV) despite the high exposure of oxygen. The overall signal decreases due to attenuation by adsorbed O. The spectra were measured at 760 eV photon energy.

References

(1) Waluyo, I.; Hunt, A. Ambient Pressure X-Ray Photoelectron Spectroscopy at the IOS (23-ID-2) Beamline at the National Synchrotron Light Source II. *Synchrotron Radiat. News* **2022**, *35* (3), 31–38. https://doi.org/10.1080/08940886.2022.2082180.

(2) Bukhtiyarov, V. I.; Hävecker, M.; Kaichev, V. V.; Knop-Gericke, A.; Mayer, R. W.; Schlögl, R. Atomic Oxygen Species on Silver: Photoelectron Spectroscopy and x-Ray Absorption Studies. *Phys. Rev. B* **2003**, *67* (23), 235422. https://doi.org/10.1103/PhysRevB.67.235422.

(3) Zangwill, A. *Physics at Surfaces*; Cambridge, 1988.

(4) Seah, M. P.; Gilmore, I. S.; Beamson, G. XPS: Binding Energy Calibration of Electron Spectrometers 5 Re-Evaluation of the Reference Energies. *Surf. Interface Anal.* **1998**, *26* (9), 642–649. https://doi.org/10.1002/(SICI)1096-9918(199808)26:9<642::AID-SIA408>3.0.CO;2-3.

(5) Rocha, T. C. R.; Oestereich, A.; Demidov, D. V.; Hävecker, M.; Zafeiratos, S.; Weinberg, G.; Bukhtiyarov, V. I.; Knop-Gericke, A.; Schlögl, R. The Silver–Oxygen System in Catalysis: New Insights by near Ambient Pressure X-Ray Photoelectron Spectroscopy. *Phys. Chem. Chem. Phys.* **2012**, *14* (13), 4554. https://doi.org/10.1039/c2cp22472k.

(6) Hsieh, M.-F.; Lin, D.-S.; Gawronski, H.; Morgenstern, K. Hard Repulsive Barrier in Hot Adatom Motion during Dissociative Adsorption of Oxygen on Ag(100). *J. Chem. Phys.* **2009**, *131* (17). https://doi.org/10.1063/1.3258849.

(7) Costina, I.; Schmid, M.; Schiechl, H.; Gajdoš, M.; Stierle, A.; Kumaragurubaran, S.; Hafner, J.; Dosch, H.; Varga, P. Combined STM, LEED and DFT Study of Ag(100) Exposed to Oxygen near Atmospheric Pressures. *Surf. Sci.* **2006**, *600* (3), 617–624. https://doi.org/10.1016/j.susc.2005.11.020.

(8) Peuckert, M. On the Adsorption of Oxygen and Potassium Hydroxide on Silver. *Surface Science* **1984**, *146* (2–3), 329–340. https://doi.org/10.1016/0039-6028(84)90434-5.

(9) Lützenkirchen-Hecht, D.; Strehblow, H. The Anodic Oxidation of Silver in 1 M NaOH: Electrochemistry, *Ex Situ* XPS and *in Situ* X-ray Absorption Spectroscopy. *Surf. Interface Anal.* **2006**, *38* (4), 686–690. https://doi.org/10.1002/sia.2188.

(10) Uhlenbrock, S.; Scharfschwerdt, C.; Neumann, M.; Illing, G.; Freund, H.-J. The Influence of Defects on the Ni 2p and O 1s XPS of NiO. *J. Phys. Condens. Matter* **1992**, *4* (40), 7973–7978. https://doi.org/10.1088/0953-8984/4/40/009.

(11) Kaspar, T. C.; Droubay, T.; Chambers, S. A.; Bagus, P. S. Spectroscopic Evidence for Ag(III) in Highly Oxidized Silver Films by X-Ray Photoelectron Spectroscopy. *J. Phys. Chem. C* **2010**, *114* (49), 21562–21571. https://doi.org/10.1021/jp107914e.

(12) Spurgeon, P. M.; Liu, D.-J.; Walen, H.; Oh, J.; Yang, H. J.; Kim, Y.; Thiel, P. A. Characteristics of Sulfur Atoms Adsorbed on Ag(100), Ag(110), and Ag(111) as Probed with Scanning Tunneling Microscopy: Experiment and Theory. *Phys. Chem. Chem. Phys.* **2019**, *21* (20), 10540–10551. https://doi.org/10.1039/C9CP01626K.
